# Diet quality indices and odds of metabolic dysfunction-associated fatty liver disease: a case-control study

**DOI:** 10.3389/fnut.2023.1251861

**Published:** 2024-01-08

**Authors:** Pushpamala Ramaiah, Kamilya Jamel Baljon, Sana A. Alsulami, Grace M. Lindsay, Lathamangeswari Chinnasamy

**Affiliations:** ^1^Faculty of Nursing, Umm Al-Qura University, Makkah, Saudi Arabia; ^2^Faculty of Nursing, Riyadh ELM University, Riyadh, Saudi Arabia

**Keywords:** alternative healthy eating index, AHEI, dietary diversity score, DDS, metabolic dysfunction-associated fatty liver disease, MASLD, dietary quality indices, DQI

## Abstract

**Objectives:**

There are only limited studies investigating the impact of dietary quality indicators, such as dietary quality index (DQI), dietary diversity score (DDS), and alternative healthy eating index (AHEI), on metabolic dysfunction-associated fatty liver disease (MASLD). Furthermore, these indicators may have different components that could lead to varying results. Therefore, this study aims to assess the nutritional quality indicators and their potential association with MASLD.

**Methods:**

The study included 128 recently diagnosed MASLD patients and 256 controls aged between 20 and 60 years. The dietary intake of participants was evaluated using a validated semi-quantitative food frequency questionnaire that consisted of 168 items. In this study, the method used to evaluate dietary diversity was based on five main food groups, specifically bread and grains, vegetables, fruits, meat, and dairy. The AHEI-2010 was computed using data collected from the FFQ.

**Results:**

After adjusting for confounders in the fully adjusted model, a significant negative correlation was observed between DDS and the risk of MASLD (OR 0.41, 95% CI 0.20, 0.97). Participants in the top quartile of AHEI had a 76% lower risk of MASLD compared with those in the bottom quartile after controlling for all potential confounders in the fully adjusted model (OR 0.24, 95% CI 0.12, 0.56).

**Conclusion:**

The results of our study suggest that there is a significant association between adherence to a high-diversity diet and a reduced likelihood of developing MASLD. Similarly, we observed a similar association between adherence to the AHEI diet and a lower risk of MASLD.

## Introduction

Metabolic dysfunction-associated fatty liver disease (MASLD) is a prevalent cause of liver abnormalities worldwide and has become increasingly common in Asia in recent years (1–3). Its global prevalence is estimated to be between 20 and 30% in Western countries and 5% and 18% in Asia (2). This disease can lead to several liver abnormalities, including non-alcoholic steatohepatitis (NASH), cirrhosis, liver failure, and liver cancer, and is believed to be the primary cause of cirrhosis of the liver (4, 5). The strong correlation between MASLD, obesity, and metabolic syndrome is well established, and the disease is also an independent risk factor for cardiovascular diseases (5, 6). Therefore, identifying risk factors for fattyliver disease is crucial. The increasing incidence of MASLD in Asia makes it particularly important to understand the risk factors associated with the disease in this region. It is essential to continue researching this disease to develop effective prevention and treatment strategies, to reduce the burden of MASLD worldwide.

Lifestyle interventions, particularly a well-balanced diet, have been found to be significantly associated with preventing and managing MASLD (7). However, it is important to understand the complete composition of dietary intake as an alternative approach to establishing a link between diet and disease (8). This method considers the collaborative and opposing interactions of different dietary components and addresses the limitation of studying individual dietary components (8). By evaluating the overall dietary pattern, researchers can examine the combined effect of multiple nutrients on health outcomes, including MASLD. It can also provide a more comprehensive understanding of the role of diet in the development and progression of MASLD. Therefore, the assessment of dietary diversity and quality, as well as the identification of specific dietary patterns, is essential in the prevention and management of MASLD. A recent study showed that higher adherence to a nutritional pattern characterized by fructose, vitamin C, vitamin A, pyridoxine, and potassium, primarily from fruits, vegetables, and nuts, is inversely associated with MASLD risk (9). Another study indicated that healthy and Western dietary patterns may be associated with the risk of MASLD. It was stated that these results can be used for developing interventions in order to promote healthy eating for the prevention of MASLD (10).

Dietary Quality Indices (DQIs) such as alternative healthy eating index (AHEI) and dietary diversity score (DDS) have been developed to provide an overall picture of chronic nutritional intake, providing insight into the characteristics of diet-related diseases (11–13). The Dietary Diversity Score (DDS) is an index used to evaluate the overall dietary intake. It is associated with a higher consumption of macronutrients and micronutrients, better diet adequacy, and higher intake of fiber, antioxidants, and other nutrients (14). Previous research has shown that a higher DDS can help reduce certain risk factors associated with MASLD, such as diabetes (15), hypertension, and cardiovascular risk factors (16). These findings suggest that a diverse diet can provide the necessary nutrients to maintain good health and reduce the risk of chronic diseases. Therefore, evaluating dietary diversity is important in developing effective strategies for preventing and managing MASLD.

To the best of our knowledge, there are only limited studies investigating the impact of dietary quality indicators, such as DQI, DDS, and AHEI, on MASLD. Furthermore, these indicators may have different components that could lead to varying results. Therefore, this study aims to assess the nutritional quality indicators and their potential association with metabolic dysfunction-associated fatty liver disease in a case–control study. By analyzing the dietary habits of individuals with and without MASLD, this study provides a better understanding of the role of diet quality in the development and prevention of the disease in this population.

## Patients and methods

### Subjects

This was a cross-sectional study conducted at Umm Al-Qura University Medical Center, Makkah, Kingdom of Saudi Arabia, in patients attending Liver and Gastroenterology Clinic. The research study enrolled 128 individuals who had been recently diagnosed with MASLD and 256 healthy controls ranging in age from 20 to 60 years old. Individuals who were diagnosed with fatty liver based on laboratory tests and liver ultrasound indicating the presence of steatosis were included in the fatty liver group. The diagnosis of fatty liver was made by a specialist doctor. MASLD was defined as elevated liver enzymes, specifically alanine aminotransferase (ALT) levels >31 mg/dl and 41 mg/dl and aspartate aminotransferase (AST) levels >31 mg/dl and 47 g/dl in women and men, respectively. On the other hand, healthy individuals who had normal laboratory test results (ALT levels <31 UI/L and 41 UI/L and AST levels < 31 UI/L and 37 UI/L in women and men, respectively) and normal liver sonography indicating no stages of hepatic steatosis were considered as the control group (17).

The study excluded participants who had specific dietary habits due to medical conditions or weight loss and those with certain medical conditions such as kidney and liver disease (e.g., viral infections, autoimmune liver disease, hemochromatosis, Wilson's disease, and alcoholic fatty liver disease), cardiovascular disease, diabetes mellitus and malignancies, thyroid disease, and autoimmune diseases. Additionally, individuals taking drugs that could harm the liver or promote weight gain were also excluded. The study also excluded participants who completed <35 items on the food frequency questionnaire or underreported/overreported their daily energy intake (<800 kcal or more than 4,500 kcal per day), but these participants were replaced with the new ones. All participants provided informed written consent before being included in the study.

### Dietary assessment

The dietary intake of the participants was evaluated using a validated semi-quantitative food frequency questionnaire that consisted of 168 items (18). Participants were asked to report their average food consumption over the past few years by selecting one of the following options: never or less than once a month, 3–4 times a month, once a week, 2–4 times a week, 5–6 times a week, once a day, 2–3 times a day, 4–5 times a day, or 6 times or more a day. The amount of each food item was converted into grams using standard household measurements, and the frequencies of consumed foods were transformed into daily intakes. The nutrient composition of all foods was determined using modified Nutritionist IV software, which provided information on EPA, DHA, sodium levels, and other micronutrients (19). Additionally, the National Nutrient Database of the US Department of Agriculture (USDA) was used in other studies to obtain daily nutrient information such as EPA and DHA for each participant (20). The N4 software output was used to obtain the sodium content of the food. The FFQ questionnaire also included an item on the salt intake of participants. The use of a standardized questionnaire and food composition tables allowed for the accurate assessment of the dietary intake of participants and ensured that the data obtained were reliable and comparable. The smoking status of the study subjects was obtained using the results of a general questionnaire that the subjects completed. Data on physical activity were obtained *via* a short form of the validated International Physical Activity Questionnaire (IPAQ), which was presented as metabolic equivalent task-minutes per week (MET-min/week).

### Dietary diversity score

The method used to evaluate dietary diversity in this study was based on the approach developed by Kant et al. (21, 22). This study considered five main food groups, which were derived from the USDA Food Guide Pyramid, specifically bread and grains, vegetables, fruits, meat, and dairy. These food groups were further divided into 23 subgroups to capture the diversity within each group. To determine the diversity score for each group, the consumption of half a serving of each subgroup was considered. Each of the five food groups could receive a maximum diversity score of 2 out of 10 (5 × 2 = 10). The total diversity score was calculated by summing up the scores from each of the five main food groups. The methodology facilitated a thorough evaluation of the extent of dietary variety and furnished significant perspectives on the general dietary patterns of the subjects.

### Alternative healthy eating index

The AHEI-2010 was computed using data collected from the FFQ. This method incorporates various aspects of the original HEI developed by Kennedy et al. (23, 24) and considers eleven components, namely, fruits, vegetables, whole grains, nuts and legumes, long-chain n-3 fats (DHA and EPA), PUFA, wine consumption, sugar-sweetened drinks and fruit juice, red and processed meats, trans-fat, and Na intake. However, since our database lacked data on wine consumption and trans-fat, only nine components were evaluated in our study. Each individual was assigned a score for each component based on their decile ranking, with the highest decile receiving a score of 10 and the lowest decile receiving a score of 1. Individuals in other deciles were given corresponding scores. Conversely, individuals with the highest intake of sugar-sweetened drinks and fruit juice, red and processed meats, and Na were given a score of 1, while those with the lowest consumption of these components were given a score of 10. The total AHEI score for each participant was then calculated by summing up the scores for these ten components, which ranged from 9 to 81. This method allowed for the evaluation of dietary quality based on multiple components and provided a comprehensive assessment of the overall dietary habits of participants.

### Statistical analysis

Statistical analyses were performed using Statistical Package for Social Sciences software version 21 (SPSS Inc., Chicago, IL, USA). The significance level was determined as *P* < 0.05. The normality of variables was checked by Kolmogorov–Smirnov and histogram tests. The study compared the general characteristics and dietary intakes of cases and controls using appropriate statistical tests such as independent sample *t*-test and χ^2^. After constructing the DDS and AHEI scores as described previously, energy-adjusted scores were obtained using the residual method (25). Participants were then categorized into quartiles based on the cutoff points obtained from the control group, and one-way ANOVA and χ^2^ tests were used to assess continuous and categorical variables across these quartiles. The association of DDS and AHEI with MASLD was evaluated using binary logistic regression in different models, with the first quartile of DDS and AHEI serving as the reference category.

## Results

The general characteristics and dietary intake of the participants are presented in Table 1. There was no significant difference between the two study groups in terms of age and gender (*p* > 0.05). However, compared with the control group, MASLD patients had a higher BMI, were more smokers, and had less physical activity (*p* < 0.05). In addition, MASLD patients had higher dietary intakes of calories, carbohydrates, fats, refined grains, high-fat dairy, and red and processed meats than controls (*p* < 0.05). The control group had higher dietary intakes of whole grains, vegetables, vitamin C, selenium, vitamin E, and vitamin A than MASLD patients (*p* < 0.05). There were no significant differences between the two groups in dietary intake.

**Table 1:** General characteristics and dietary intake among the study groups.

**Variables**	**Controls (*n =* 256)**	**MASLD (*n =* 128)**	*P*-value^*^
Age (years)	38.1 ± 7.9	37.6 ± 7.7	0.325
Male, *n* (%*)*	133 (51.8)	71 (55.6)	0.258
BMI (Kg/m^2^)	25.3 ± 2.9	30.2 ± 3.7	< 0.001
Smoking, *n* (%)	7 (2.7)	9 (7.1)	0.005
Physical activity (MET-min/week)	1590 ± 949	1119 ± 616	< 0.001
**Dietary intake**			
Energy intake (kcal/day)	2117 ± 594	2325 ± 614	0.006
Carbohydrate (gr)	317 ± 27	387 ± 41	0.041
Protein (gr)	67 ± 15	63 ± 12	0.145
Fat (gr)	66 ± 17	82 ± 14	0.037
Fiber (gr/1000 Kcal)	16.4 ± 7.3	16.1 ± 6.2	0.164
Whole grains (gr/day)	58.0 ± 91.3	41.6 ± 58.2	0.041
Refined grains (gr/day)	322 ± 145	396 ± 188	< 0.001
High fat dairy products (gr/day)	84.4 (38.9 – 167.5)	231.5 (109.6 – 310.8)	< 0.001
Fruits (gr/day)	311 ± 244	293 ± 214	0.064
Vegetables (g/day)	302 ± 134	261 ± 125	0.001
Nuts and legume (gr/day)	15.4 (9.2 – 24.7)	14.8 (8.7 – 28.3)	0.412
Red and processed meat (gr/day)	21.2 ± 12.4	27.1 ± 16.8	0.001
Vitamin D (μg/day)	2.7 ± 2.4	2.0 ± 1.8	0.092
Vitamin C (mg/dar)	57.3 ± 24.2	42.8 ± 19.0	0.042
Calcium (mg/day)	1216.3 ± 323.3	993.1 ± 311.1	0.084
Zinc (mg/day)	246.3 ± 86.3	229.5 ± 84.1	0.088
Magnesium (mg/day)	8.9 ± 3.1	9.1 ± 4.4	0.127
Iron (mg/day)	9.4 ± 3.6	9.2 ± 3.5	0.732
Selenium (μg/day)	17.1 ± 8.6	10.0 ± 6.6	0.048
Vitamin E (IU/day)	2.24 ± 1.6	1.49 ± 1.4	0.031
Vitamin A (IU/day)	788.6 ± 351.2	541.4 ± 341.2	0.042
Vitamin B2 (IU/day)	2.1 ± 1.2	2.0 ± 1.2	0.113
Vitamin B6 (mg/day)	2.4 ± 1.2	2.3 ± 1.3	0.788
DDS scores	5.0 ± 1.7	4.7 ± 1.4	0.11
AHEI scores	50.4 ± 8.8	45.5 ± 9.6	< 0.001

MET, metabolic equivalent task; DDS, dietary diversity score; AHEI, alternative healthy eating index; MASLD, metabolic dysfunction-associated fatty liver disease; IU, international unit; BMI, body mass index. Data are presented as mean ± SD for continuous variables and numbers and percentages for categorical variables.

* Independent sample t-test and chi-square were used for continuous and categorical variables, respectively.

Table 2 presents the dietary intakes of cases and controls in various quartiles of DDS and AHEI scores. Individuals in the top quartile of DDS had higher consumption of fruits, vegetables, carbohydrates, protein, fiber, whole grains, nuts, and legumes when compared with those in the bottom quartile. Moreover, those in the fourth quartile of AHEI had lower intakes of fat, refined grains, high-fat dairy products, and red and processed meat in comparison to participants in the first quartile. No other significant differences were observed in terms of dietary intakes across quartiles of DDS and AHEI scores.

**Table 2:** Dietary and nutrient intakes of study participants across quartiles (Q) of DDS and AHEI scores.

	**Quartiles of DDS**					**Quartiles of AHEI**				
	**Q1**	**Q2**	**Q3**	**Q4**	***P*** **-value** ^†^	**Q1**	**Q2**	**Q3**	**Q4**	***P*** **-value** ^†^
Age (year)	36.9 ± 8.6	38.0 ± 8.5	39.4 ± 9.2	37.2 ± 7.3	0.322	37.8 ± 8.1	37.0 ± 7.9	38.2 ± 8.3	37.8 ± 7.5	0.147
Male (%)	53.8	51·4	49·3	56.3	0.188	54·4	52·5	56·9	53·4	0.454
BMI(Kg/m^2^)	27.1 ± 4.1	26.7 ± 4.4	26.8 ± 4.4	26.8± 3.9	0.504	26.2 ± 3.1	27.5 ± 4.2	26.2 ± 3.4	27.4 ± 4.1	0.413
Smoking, (%)	4.9	5.1	4.7	5.2	0.522	4.7	4.8	5.6	5.1	0.427
Physical activity (MET-min/week)	1445 ± 848	1456 ± 811	1387 ± 885	1434 ± 874	0.565	1437 ± 878	1411 ± 871	1429 ± 805	1501 ± 985	0.139
**Dietary intake**										
Energy intake (kcal/day)	2323 ± 601	2198 ± 624	2287 ± 641	2317 ± 658	0.357	2273 ± 649	2235 ± 619	2316 ± 653	2246 ± 618	0.369
Carbohydrate (gr)	51.1 ± 7.7	57.7 ± 6.7	59.0 ± 6.3	60.1 ± 6.8	< 0.001	51.1 ± 7.2	52.1 ± 7.5	55.6 ± 6.4	60.2 ± 6.7	< 0.001
Protein (gr)	13.1 ± 2.4	13.5 ± 2.1	13.8 ± 2.5	14.5 ± 2.5	< 0.001	13.3 ± 1.8	13.4 ± 2.3	14.0 ± 2.1	14.3 ± 2.5	< 0.001
Fat (gr)	33.4 ± 7.9	31.2 ± 6.4	30.1 ± 5.8	29.8 ± 5.7	< 0.001	32.4 ± 7.2	31.5 ± 6.1	29.6 ± 5.8	28.8 ± 5.4	< 0.001
Fiber (gr/1000 kcal)	14.8 ± 7.9	16.3 ± 6.7	17.3 ± 6.5	17.9 ± 6.8	< 0.001	13.2 ± 4.2	13.9 ± 5.8	14.8 ± 6.5	15.5 ± 6.9	0.031
Whole grains (gr/day)	22.7 ± 17.5	45.1 ± 34.9	84.0 ± 53.7	97.0 ± 73.7	< 0.001	31.7 ± 17.5	52.1 ± 34.9	96.0 ± 93.7	107.0 ± 83.4	< 0.001
Refined grains (gr/day)	394 ± 194	341 ± 135	262 ± 144	241 ± 124	< 0.001	402 ± 188	352 ± 134	282 ± 137	253 ± 125	< 0.001
High-fat dairy products (gr/day)	178 ± 154	151 ± 135	142 ± 114	138 ± 98	0.005	191 ± 149	177 ± 122	156 ± 147	152 ±102	0.008
Fruits (gr/day)	210 ± 146	332 ± 225	422 ± 247	453 ± 261	< 0.001	194 ± 94	287 ± 118	320 ± 209	408 ± 219	< 0.001
Vegetables (gr/day)	250 ± 133	301 ± 139	316 ± 156	341 ± 164	0.016	231 ± 121	252 ± 127	289 ± 148	305 ± 151	0.028
Nuts and legume (gr/day)	15.8 ± 12.4	20.5 ± 15.7	28.4 ± 25.8	33.1 ± 25.8	< 0.001	18.8 ± 12.4	28.5 ± 15.7	31.4 ± 20.1	38.4 ± 21.8	< 0.001
Red and processed meat (gr/day)	25.1 ± 19.3	23.7 ± 19.2	20.4 ± 15.2	19.8 ± 13.2	0.042	31.2 ± 21.3	28.4 ± 19.2	25.4 ± 14.2	22.4 ± 13.1	0.031

Data are presented as mean ± SD for continuous variables and numbers and percentages for categorical variables.

† Obtained from ANOVA.

Table 3 shows the multivariable-adjusted OR and 95% CI for MASLD across quartiles of DDS and AHEI. After adjusting for age, sex, body mass index, smoking, and physical activity, a significant negative correlation was observed between DDS and the risk of MASLD (OR 0.44, 95% CI 0.19, 0.94). This finding remained consistent after further controlling for dietary energy intake, with participants in the highest quartile of DDS being 59% less likely to have MASLD than those in the lowest quartile (OR 0.41, 95% CI 0.20, 0.97). Additionally, a significant inverse association was found between AHEI and the odds of MASLD. Participants in the top quartile of AHEI had a 76% lower risk of MASLD compared with those in the bottom quartile after controlling for all potential confounders in the fully adjusted model (OR 0.24, 95% CI 0.12, 0.56).

**Table 3:** Risk for MASLD according to quartiles (Q) of DDS and AHEI scores (odds ratios and 95 % confidence intervals)^#^.

	**Quartiles of DDS**					**Quartiles of AHEI**				
	**Q1**	**Q2**	**Q3**	**Q4**	**P-trend**	**Q1**	**Q2**	**Q3**	**Q4**	**P-trend**
Crude model	1	1.47 (0.84, 2.56)	0.73 (0.39, 1.36)	0.55 (0.28, 1.08)	0.021	1	0.55 (0.31, 0.96)	0.24 (0.12, 0.47)	0.41 (0.23, 0.74)	0.011
Model 1^*^	1	1.49 (0.85, 2.60)	0.72 (0.39, 1.35)	0.55 (0.28, 1.07)	0.034	1	0.55 (0.32, 0.96)	0.23 (0.12, 0.47)	0.41 (0.22, 0.74)	0.032
Model 2^†^	1	1.33 (0.69, 2.58)	0.67 (0.32, 1.37)	0.44 (0.19, 0.94)	0.018	1	0.47 (0.24, 0.91)	0.19 (0.08, 0.42)	0.25 (0.12, 0.55)	0.004
Model 3^‡^	1	1.36 (0.70, 2.64)	0.68 (0.33, 1.41)	0.41 (0.20, 0.97)	0.011	1	0.48 (0.25, 0.93)	0.19 (0.08, 0.43)	0.24 (0.12, 0.56)	< 0.001

# Obtained from regression logistic.

* Model 1: adjusted for age and sex.

† Model 2: adjusted for model 1 and body mass index, smoking, and physical activity.

‡ Model 3: adjusted for model 1 and body mass index, smoking, physical activity, and dietary intake of energy.

## Discussion

Our study revealed that there is a negative relationship between the Dietary Diversity Score (DDS) and the risk of MASLD. We took into account several variables that could potentially affect this relationship, such as age, sex, body mass index, smoking, physical activity, and energy intake, and found an inverse association between DDS and MASLD. We also observed a similar inverse relationship between AHEI and MASLD, both before and after controlling for the aforementioned confounders.

Numerous studies have shown that an overall dietary pattern, rather than focusing on individual foods or nutrients, is a better predictor of overall diet quality and is associated with various diseases. In particular, DDS and AHEI have been used as indicators of diet quality in relation to non-communicable diseases such as cardiovascular disease, polycystic ovaries, and some cancers (26–29). In the present study, a high DDS was found to be associated with decreased odds of MASLD. This finding is consistent with previous research that has shown an inverse association between DDS and the risk of diabetic nephropathy (29) and bladder cancer (30). In another study, by emphasizing the higher diversity scores for vegetables and fewer diversity scores for meat and refined grains, high DDS is inversely correlated with the risk of MASLD (15).

One possible explanation for the protective association of a diverse diet against MASLD is its high nutrient content, including antioxidants. Several studies have demonstrated an inverse association between dietary intake of antioxidants and the risk of MASLD (31, 32). Additionally, diets with a high DDS tend to be rich in fruits and vegetables, which have also been shown to be protective against MASLD (33). Such diets are also typically high in fiber, which can reduce the risk of MASLD (34, 35). However, it should be noted that not all studies have found a significant association between dietary diversity and the risk of certain diseases, such as breast cancer (36). In some cases, confounding factors may not have been adequately adjusted to determine the independent association between dietary diversity and disease risk. Overall, increasing the diversity score of one's diet by emphasizing higher diversity scores for vegetables and fewer diversity scores for meat and refined grains may be beneficial to managing MASLD (15).

The current study found that a higher level of adherence to the AHEI was associated with a reduced likelihood of MASLD. Previous research has also shown that healthy dietary patterns are associated with a lower risk of MASLD, while Western dietary patterns are linked to an increased risk of this disease (10). In a large population-based study in Germany, individuals with the highest AHEI scores were less likely to develop colorectal cancer compared with those with the lowest scores (37). In a meta-analysis of observational studies, a high adherence to the AHEI dietary pattern was associated with a reduced risk of all-cause mortality and cardiovascular and cancer mortality (38).

Additionally, a study found that AHEI scores were significantly higher among individuals with high levels of sensitive C-reactive protein (Hs-CRP), which is a marker of inflammation in the body (39). A systematic review and meta-analysis of observational studies also showed that high adherence to the AHEI was associated with a reduced risk of cancer mortality (38). Fruit consumption has also been shown to play a preventive role in the incidence of MASLD (33, 40). Furthermore, dietary intake of antioxidants is higher among individuals who have greater adherence to the AHEI (41). A high AHEI score is also associated with a lower intake of red and processed meats, which are known risk factors for MASLD (42, 43).

This study has several limitations that should be taken into consideration when interpreting the results. First, the study design was a case–control study, which is prone to several types of bias, including selection and recall bias. Although case–control studies are cost-effective and quick to conduct, they are highly vulnerable to bias. Recall bias is a particular concern in this study, as cases may recall their previous diet differently in light of their MASLD diagnosis. Additionally, dietary assessment occurred after diagnosis, which can further exacerbate recall bias. It is also possible that cases may have altered their diet before diagnosis due to early symptoms of the disease, leading to attenuation of the estimates. However, it is worth noting that MASLD-related symptoms may prompt patients to consume healthier foods rather than unhealthy ones. Furthermore, with all epidemiological studies that use food frequency questionnaire (FFQ), misclassification of study participants is unavoidable. Despite the efforts of the researchers to control for several confounding factors, the possibility of residual confounding cannot be entirely ruled out. It is important to acknowledge these limitations when interpreting the findings of this study and consider them in any future research in this area.

## Conclusion

The results of our study suggest that there is a significant association between adherence to a high-diversity diet and a reduced likelihood of developing MASLD. Specifically, our findings indicate that individuals who closely followed a diverse diet had lower odds of developing MASLD. Similarly, we observed a similar association between adherence to the AHEI diet and a lower risk of MASLD. Participants who had greater adherence to the AHEI diet were found to have a lower likelihood of developing MASLD compared with those who had the lowest adherence to this dietary pattern. These results highlight the importance of consuming a diverse and healthy diet in reducing the risk of MASLD.

## Data availability statement

The raw data supporting the conclusions of this article will be made available by the authors, without undue reservation.

## Ethics statement

Ethical approval of this study was obtained from the Ethical Committee of Umm Al-Qura University (ID: HAPO-02-K-012-2023-02-345) Makkah, Kingdom of Saudi Arabia. The studies were conducted in accordance with the local legislation and institutional requirements. The participants provided their written informed consent to participate in this study.

## Author contributions

PR and KJ conceptualized and designed the study and supervised the project. SA participated in the data acquisition and literature review of the articles. GL analyzed and interpreted the data. PR, KJ, and LC drafted the initial manuscript. All authors approved the final version of the manuscript.
